# *De Novo* Generation and Characterization of New Zika Virus Isolate Using Sequence Data from a Microcephaly Case

**DOI:** 10.1128/mSphereDirect.00190-17

**Published:** 2017-05-17

**Authors:** Yin Xiang Setoh, Natalie A. Prow, Nias Peng, Leon E. Hugo, Gregor Devine, Jessamine E. Hazlewood, Andreas Suhrbier, Alexander A. Khromykh

**Affiliations:** a Australian Infectious Diseases Research Centre, School of Chemistry and Molecular Biosciences, University of Queensland, St. Lucia, Queensland, Australia; b Inflammation Biology Group, QIMR Berghofer Medical Research Institute, Brisbane, Queensland, Australia; c Mosquito Control Group, QIMR Berghofer Medical Research Institute, Brisbane, Queensland, Australia; Boston University School of Medicine; Icahn School of Medicine at Mount Sinai; National Institute of Allergy and Infectious Diseases

**Keywords:** infectious DNA, Zika virus, flavivirus, mouse model

## Abstract

Zika virus (ZIKV) has recently emerged and is the etiological agent of congenital Zika syndrome (CZS), a spectrum of congenital abnormalities arising from neural tissue infections *in utero*. Herein, we describe the *de novo* generation of a new ZIKV isolate, ZIKV_Natal_, using a modified circular polymerase extension reaction protocol and sequence data obtained from a ZIKV-infected fetus with microcephaly. ZIKV_Natal_ thus has no laboratory passage history and is unequivocally associated with CZS. ZIKV_Natal_ could be used to establish a fetal brain infection model in IFNAR^−/−^ mice (including intrauterine growth restriction) without causing symptomatic infections in dams. ZIKV_Natal_ was also able to be transmitted by Aedes aegypti mosquitoes. ZIKV_Natal_ thus retains key aspects of circulating pathogenic ZIKVs and illustrates a novel methodology for obtaining an authentic functional viral isolate by using data from deep sequencing of infected tissues.

**IMPORTANCE** The major complications of an ongoing Zika virus outbreak in the Americas and Asia are congenital defects caused by the virus’s ability to cross the placenta and infect the fetal brain. The ability to generate molecular tools to analyze viral isolates from the current outbreak is essential for furthering our understanding of how these viruses cause congenital defects. The majority of existing viral isolates and infectious cDNA clones generated from them have undergone various numbers of passages in cell culture and/or suckling mice, which is likely to result in the accumulation of adaptive mutations that may affect viral properties. The approach described herein allows rapid generation of new, fully functional Zika virus isolates directly from deep sequencing data from virus-infected tissues without the need for prior virus passaging and for the generation and propagation of full-length cDNA clones. The approach should be applicable to other medically important flaviviruses and perhaps other positive-strand RNA viruses.

## Abstract (precis)

The major complications of an ongoing Zika virus outbreak in the Americas and Asia are congenital defects caused by the virus’s ability to cross the placenta and infect the fetal brain. The ability to generate molecular tools to analyze viral isolates from the current outbreak is essential for furthering our understanding of how these viruses cause congenital defects. The majority of existing viral isolates and infectious cDNA clones generated from them have undergone various numbers of passages in cell culture and/or suckling mice, which is likely to result in the accumulation of adaptive mutations that may affect viral properties. The approach described herein allows rapid generation of new, fully functional Zika virus isolates directly from deep sequencing data from virus-infected tissues without the need for prior virus passaging and for the generation and propagation of full-length cDNA clones. The approach should be applicable to other medically important flaviviruses and perhaps other positive-strand RNA viruses.

## INTRODUCTION

Zika virus (ZIKV) is a mosquito-borne flavivirus that has recently reemerged, with transmission now reported in >70 countries and territories (1). In 2016, the World Health Organization declared the ZIKV pandemic a public health emergency of international concern (2). In humans, an estimated 80% of primary infections with ZIKV appear to be asymptomatic (3), with the majority of symptomatic cases showing mild disease (including rash, conjunctivitis, arthralgia, myalgia, and fever), although infection can occasionally lead to Guillain-Barré syndrome (1, 3, 4). The primary concern is infection of pregnant mothers, which can lead to the virus infecting (and thereby damaging) the fetal brain, resulting in a range of congenital birth defects now recognized as congenital Zika syndrome (CZS) (1, 3). In a recent study of symptomatic mothers, abnormal clinical or brain imaging findings were seen in 55% of the infants born to mothers infected in the first trimester, 52% of the infants born to mothers infected in the second trimester, and 29% of the infants born to mothers infected in the third trimester (5). In that study, microcephaly was evident in 3.4% of the neonates examined, with microcephaly usually associated with fetal growth restriction. There is also emerging evidence that children who were infected *in utero* but showed no overt congenital defects at birth manifest a range of disabilities over time (1, 5, 6).

One of the first cases of ZIKV-associated severe microcephaly was identified in 2015 in an aborted fetus from a mother who was infected with ZIKV during the first trimester of pregnancy while living in the Natal region of Brazil (7). Viral RNA, viral proteins, and viral particles were detected in fetal brain tissues (7), and a complete genome sequence of the virus was obtained by next-generation sequencing of brain tissue (GenBank accession number KU527068). This ZIKV_Natal_ sequence therefore represents a ZIKV sequence unequivocally associated with a human case of microcephaly and is free of any potential adaptive mutations arising from serial passage, for instance, in cells *in vitro* or in suckling mice.

Flavivirus infectious cDNA clones have facilitated numerous scientific discoveries over the years; however, the well-documented instability of plasmids harboring full-length flavivirus cDNA sequences during propagation in bacteria remains an issue. Recently, three groups have independently reported that the toxicity of ZIKV sequences has hindered the stable propagation in bacteria of plasmid DNAs harboring full-length ZIKV cDNA (8–10), with insertion of introns required to resolve the issue (9, 10).

We have recently developed and optimized a protocol to generate *de novo* infectious flaviviruses that does not involve the generation of DNA plasmids encoding the complete viral genome or *in vitro* RNA transcription. The method, based on the circular polymerase extension cloning protocols described previously (11), significantly simplifies and accelerates the process of *de novo* virus generation (12, 13). The protocol involves the generation of a circular DNA (with high-fidelity polymerase) that encompasses the entire viral cDNA sequence *in vitro*. A cytomegalovirus (CMV) promoter sits directly upstream of the first nucleotide of viral cDNA, and following the transfection of circular polymerase extension reaction (CPER) products into mammalian cells, viral RNA is synthesized and infectious virus is recovered. The protocol has been successfully used to generate wild-type (WT) and mutant West Nile viruses (13), chimeric viruses consisting of different West Nile virus strains (12), and chimeric viruses consisting of West Nile virus and Brazilian Rocio virus (14).

Herein, we describe the application of a modified CPER protocol to generate an infectious ZIKV_Natal_ isolate *de novo* from a published sequence. We describe the behavior of this new isolate *in vitro*, in mouse models, and in Aedes aegypti mosquitoes.

## RESULTS

### Recovery of infectious ZIKV_Natal_ from the published sequence.

**FIG 1:**
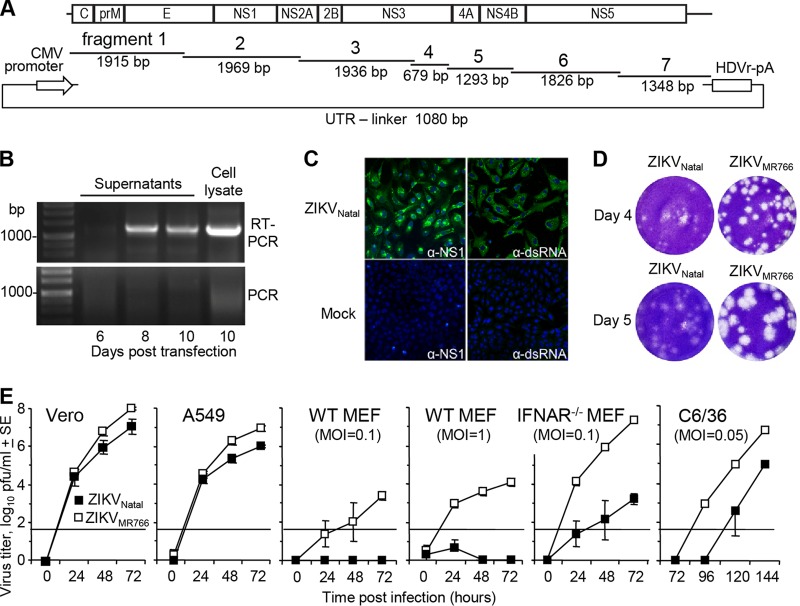
*De novo* generation of infectious ZIKV_Natal_ by CPER. (A) Schematic representation of CPER assembly with seven synthetic DNA fragments covering the entire genome of ZIKV_Natal_ with ~22-nt overlapping ends. The fragments were mixed with the UTR linker, which contained the CMV promoter followed by the first 22 nt of ZIKV_Natal_ and, at the other end, the last 22 nt of ZIKV_Natal_, an HDVr site, and a poly(A) tail (pA). After CPER, the products were transfected into Vero cells. (B) On the posttransfection days indicated, RNA was isolated from Vero cell supernatants and a cell lysate and subjected to RT-PCR with fragment 5-specific primers (RT-PCR). To demonstrate that the bands were not due to contaminating DNA, a control (RT minus) PCR was performed in parallel (PCR). (C) Vero cells were infected with ZIKV_Natal_ (culture supernatant from day 10 posttransfection), and mock-infected cells were used as controls. At 3 days postinfection, Vero cells were analyzed by immunofluorescent-antibody staining with anti-NS1 (4G4) and anti-dsRNA (3G1) antibodies (green) and DAPI counterstain (blue). (D) Plaque morphology following infection of Vero cells with ZIKV_Natal_ and ZIKV_MR766_. Vero cells were fixed and stained with crystal violet on the postinfection days indicated. (E) Growth kinetics of ZIKV_Natal_ and ZIKV_MR766_ in the cell lines indicated. Infection was performed at an MOI of 0.1, unless otherwise indicated. Virus titers in the culture supernatants were determined by plaque assays on Vero cells. The data and standard errors (SE) shown are from six independent experiments with Vero cells, A549 cells, and WT MEFs (MOI of 1) and three independent experiments with WT MEFs (MOI of 0.1), IFNAR^−/−^ MEFs, and C6/36 cells. The horizontal line represents the limit of detection (50 PFU/ml).

The sequence of ZIKV_Natal_ (derived by deep sequencing of an infected fetal brain [7]) was obtained from GenBank (accession number KU527068). Seven overlapping double-stranded DNA (dsDNA) fragments covering the entire viral sequence (Fig. 1A) were cloned into pUC19 vectors. An eighth pUC19 plasmid contained the untranslated region (UTR) linker, which comprised the minimal CMV promoter and the first 22 nucleotides (nt) of the viral sequence at one end and the last 22 nt of the viral sequence, the hepatitis delta virus ribozyme (HDVr) site, and a polyadenylation (pA) signal at the other (Fig. 1A). The CMV promoter initiates viral RNA transcription, and the HDVr sequence ensures authentic formation of the 3′ UTR. These plasmids were then used to generate cDNA fragments by PCR, with the resulting eight dsDNA fragments mixed in equimolar amounts and subjected to 12 cycles of CPER with Q5 DNA polymerase. The CPER products were then transfected into Vero cells, and on days 6, 8, and 10 after transfection, the tissue culture supernatants were harvested. The presence of viral RNA in the supernatants was demonstrated by reverse transcription (RT)-PCR of isolated RNA with fragment 5-specific primers, with the minus RT control showing no bands (Fig. 1B). Further confirmation of virus recovery was obtained by immunofluorescent-antibody staining of Vero cells with anti-NS1 and anti-dsRNA antibodies 3 days after infection with the day 10 supernatant (Fig. 1C).

The viral titer in the day 10 supernatant was 5 × 10^5^ PFU/ml, as determined by plaque assay on Vero cells. This virus (passage 0) was amplified in C6/36 cells in two independent expansions to produce passage 1 virus stocks that were used in subsequent experiments, i.e., (i) infection at a multiplicity of infection (MOI) of 0.001, reaching 7.6 log_10_ 50% cell culture infective doses (CCID_50_) by day 5, and (ii) infection at an MOI of 0.003, reaching 4.5 × 10^7^ PFU/ml on day 6 and 1.25 × 10^8^ PFU/ml on day 8. In a separate experiment, a different DNA polymerase, PrimeSTAR GXL, was used to generate circular DNA by CPER. Transfection of this DNA resulted in the recovery of higher virus titers by day 10 after transfection (passage 0, 2.6 × 10^7^ PFU/ml). These results illustrate that the modified CPER protocol can efficiently produce high titers of ZIKV either directly after transfection or after a single passage.

### Sequencing of ZIKV_Natal_.

RNA from virus recovered from day 10 supernatants (Fig. 1B) was used to generate amplicons by RT-PCR, which were then subjected to deep sequencing with the Illumina MiSeq platform. A total of 451,583 reads were mapped to 100% of the genome, with a mean read coverage of 5,734. The sequencing quality scores were 83.4% at Q20 and 76.2% at Q30. The only changes from the consensus published sequence (accession no. KU527068) were polymorphisms at nt 4328 at the start of the NS2B gene (C to T; a read coverage, 9,170, 83% T, 14% C); nt 4574 at the end of the NS2B gene (A to G; read coverage, 10,322; 98% G); and nt 5900 in the NS3 gene (C to T; read coverage, 3,960; 47% C, 51% T). None of these nucleotide changes introduced amino acid changes. The remaining sequence matched the published sequence.

### *In vitro* growth properties of recovered ZIKV_Natal_ compared with those of the prototype African MR766 isolate.

The plaque morphology of ZIKV_Natal_ was compared with that of the prototype mouse-adapted African isolate ZIKV_MR766_ in Vero cells. The plaques generated by ZIKV_Natal_ were generally smaller and less distinct than those produced by ZIKV_MR766_ (Fig. 1D).

Analysis of growth kinetics revealed that ZIKV_Natal_ replicated less efficiently than ZIKV_MR766_ in Vero, A549, and C6/36 cells and in IFNAR^−/−^ mouse embryonic fibroblasts (MEFs) infected at an MOI of 0.1 (determined on the basis of viral titers in Vero cells) (Fig. 1E). ZIKV_Natal_ was unable to replicate efficiently in WT MEFs infected at an MOI of 0.1 or 1 on the basis of viral titers determined in Vero cells (Fig. 1E, WT MEFs). In contrast, ZIKV_MR766_ did replicate in these cells, perhaps reflecting the fact that ZIKV_MR766_ has been mouse adapted, with more than 100 serial passages in mouse brains (15, 16). ZIKV_MR766_ also grew substantially better in IFNAR^−/−^ MEFs than ZIKV_Natal_ did (Fig. 1E, IFNAR^−/−^ MEFs).

### ZIKV_Natal_ infection of WT, IFNAR^−/−^, and IRF7^−/−^ mice.

ZIKV infection of WT mice generally does not produce detectable viremia (17). We similarly were unable to detect viremia in WT (C57BL/6J) mice after subcutaneous (s.c.) infection with ZIKV_MR766_ or ZIKV_Natal_ by CCID_50_ assays. Even intravaginal infection (18) with a dose of 6 log_10_ CCID_50_ of ZIKV_Natal_ failed to produce detectable viremia (data not shown).

**FIG 2:**
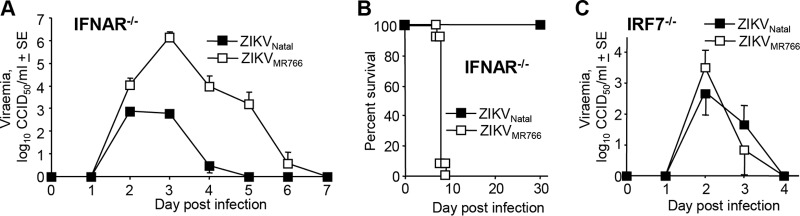
ZIKV_Natal_ infection of IFNAR^−/−^ and IRF7^−/−^ mice. (A) Viremia in IFNAR^−/−^ mice (female, 8 to 12 weeks old) after infection with ZIKV_Natal_ or ZIKV_MR766_ (s.c., 3 log_10_ CCID_50_), determined by CCID_50_ assays (*n* = 5 for ZIKV_Natal_, *n* = 11 for ZIKV_MR766_). Limit of detection, 2 log_10_ CCID_50_. (B) Survival of IFNAR^−/−^ mice (female, 8 to 12 weeks old) after s.c. infection with ZIKV_Natal_ or ZIKV_MR766_. The dose of ZIKV_MR766_ was 10^3^ CCID_50_ (*n* = 13). A range of viral doses of ZIKV_Natal_ were tested, i.e., 10^3^ CCID_50_ (*n* = 8), 10^4^ CCID_50_ (*n* = 5), 10^5^ CCID_50_ (*n* = 5), and 10^6^ CCID_50_ (*n* = 6), with no mice requiring euthanasia. (C) Viremia in IRF7^−/−^ mice (female, 8 to 12 weeks old) after infection with ZIKV_Natal_ or ZIKV_MR766_ (s.c., 3 log_10_ CCID_50_) (*n* = 3 to 5). SE, standard error.

A number of mouse ZIKV models have used IFNAR^−/−^ mice (17), and ZIKV_Natal_ (10^3^ CCID_50_ s.c.) produced a 4- to 5-day viremia in IFNAR^−/−^ mice that was ~1 to 4 logs lower than that produced by infection with the same dose of ZIKV_MR766_ (Fig. 2A). Increasing the s.c. ZIKV_Natal_ infection dose to 6 log_10_ CCID_50_ increased the peak viremia by 2 to 3 logs (see Fig. S1A in the supplemental material).

10.1128/mSphereDirect.00190-17.1 FIG S1 (A). ZIKV_Natal_ infection (s.c., 6 log_10_ CCID_50_) of IFNAR^−/−^ and IRF7^−/−^ mice (>7 weeks old). No clinical symptoms were observed. *n* = 5 per group (B). Survival of IFNAR^−/−^ mice after ZIKV_Natal_ infection of young mice and i.p. inoculation (*n* = 3 for 4-week-old mice; *n* = 5 for the remaining groups). Mice were euthanized when hind leg weakness reached an ethically defined level of severity. Groups with deaths also showed 1 to 2 days of mild hunching (C). Infection of male IFNAR^−/−^ mice (8 to 12 weeks old) with 5 log_10_ CCID_50_ of ZIKV_Natal_ s.c. (*n* = 3). Testes were tested on day 7, and the titer is indicated by the red data point. Download FIG S1, TIF file, 0.5 MB. Copyright © 2017 Setoh et al. 2017 Setoh et al. This content is distributed under the terms of the Creative Commons Attribution 4.0 International license.

ZIKV_MR766_ infection of IFNAR^−/−^ mice was always highly symptomatic, and all mice reached ethically defined endpoints requiring euthanasia by days 7 to 9 (Fig. 2B), consistent with previous reports (17, 19). In contrast, s.c. ZIKV_Natal_ infection of female IFNAR^−/−^ mice (>8 weeks of age) with a range of viral inoculation doses (3 to 6 log_10_ CCID_50_) resulted in a 100% survival rate, with no animals displaying any symptoms (Fig. 2B). Nevertheless, ZIKV_Natal_ could produce symptomatic infections in IFNAR^−/−^ mice requiring euthanasia in 30 to 40% of the animals when (i) 4-week-old IFNAR^−/−^ mice were infected s.c. with 3 log_10_ CCID_50_ (consistent with age-dependent susceptibility in flavivirus models [20]) or when (ii) 8- to 12-week-old mice were infected intraperitoneally with 5 log_10_ CCID_50_ (see Fig. S1B).

**FIG 3:**
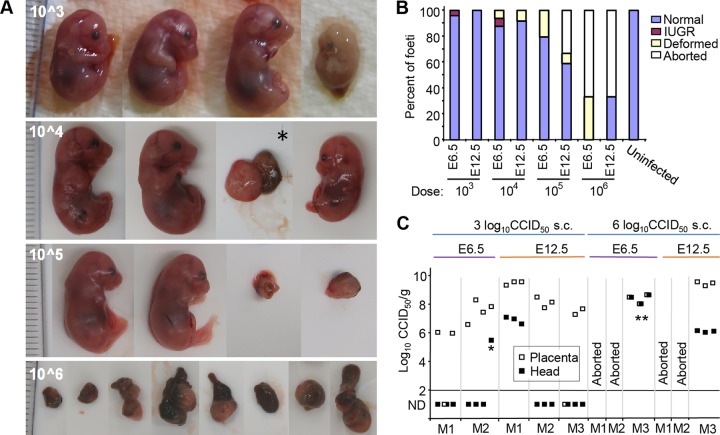
Pregnancy outcomes of ZIKV_Natal_-infected IFNAR^−/−^ dams. (A) Examples of E17.5/E18.5 fetuses from IFNAR^−/−^ × IFNAR^−/−^ mating after s.c. infection of dams (>8 weeks of age) at E6 (or E12.5 [*]) with the ZIKV_Natal_ doses indicated. Fetuses with clear signs of IUGR are shown facing left. *, Severely deformed fetus and placenta. Severely deformed fetal/placental masses are also shown in the right two images in row 10^5 and all of the images in row 10^6. (B) The percentages of fetuses that appeared normal, showed IUGR, and were severally deformed are shown. Data are from three pregnancies per group (mean of 7.78 ± 1.55 [standard deviation] fetuses per pregnancy). White bars indicate that one (33%) or two (66%) of the dams aborted. (C) Placenta and head virus titers of fetuses after large- and small-dose ZIKV_Natal_ infection of dams at E6 or E12.5. *, Fetus with IUGR shown at the top right of panel A; **, severally deformed fetal/placental masses. ND, not detected.

ZIKV_Natal_ and ZIKV_MR766_ infections of IRF7^−/−^ mice were also examined, with both viruses producing detectable viremia (Fig. 2C). Although mild hunching was observed for 1 to 2 days after ZIKV_MR766_ infection, no IRF7^−/−^ mice required euthanasia after s.c. infection with either virus at 3 log_10_ CCID_50_. Infection of IRF7^−/−^ mice with increasing s.c. doses of ZIKV_Natal_ ranging from 3 (Fig. 3C) to 6 log_10_ CCID_50_ brought forward but did not significantly increase the peak viremia, and the mice did not show any symptoms (see Fig. S1A).

### ZIKV_Natal_ fetal infection of IFNAR^−/−^ dams.

Highly symptomatic infection of dams complicates the ability to establish an ethically acceptable fetal ZIKV infection model. As s.c. ZIKV_Natal_ infection of IFNAR^−/−^ mice >8 weeks old was asymptomatic, these conditions were used to infect pregnant dams at embryonic day 6.5 (E6.5; early pregnancy) and E12.5 (mid pregnancy), nominally the first and second trimesters (21), with a range of ZIKV_Natal_ doses. Dams and fetuses were euthanized at E17.5/E18.5, and (i) fetuses were photographed (Fig. 3A), (ii) the pregnancy outcomes were quantitated (Fig. 3B), and (iii) the viral tissue titers in the placentas and heads of selected fetuses were determined by CCID_50_ assays (Fig. 3C). Intrauterine growth restriction (IUGR) (described previously in mice [18, 22] and humans [5]) was observed at doses of 3 to 4 log_10_ CCID_50_ and infection at E6.5 (Fig. 3A and B, fetuses facing left). With larger doses (4 to 6 log_10_ CCID_50_), severely deformed fetal/placental masses were evident (Fig. 3A), with such outcomes and abortions (reported previously [21]) increasing with the inoculation dose (Fig. 3B).

Most placentas contained high titers of virus after inoculation with large or small doses of ZIKA_Natal_ at either E6.5 or E12.5 (Fig. 3C). As might be expected, the head of a fetus with IUGR and the deformed fetal/placental masses contained infectious ZIKV (Fig. 3C). The heads of some outwardly normal fetuses were also infected, with titers 2 to 3 logs lower than the corresponding placental titers (Fig. 3C). At the 3 log_10_ CCID_50_ dose, many head titers were below the limit of detection, which was not the case at 6 log_10_ CCID_50_ (Fig. 3C).

Infection of IRF7^−/−^ dams at E6.5 with a s.c. dose of 4 or 6 log_10_ CCID_50_ of ZIKV_Natal_ did not result in detectable replicating virus in the placentas or the fetal heads (*n* = 3 for each dose). Male IFNAR^−/−^ mice could also be infected, with the testes becoming infected (see Fig. S1C), consistent with previous reports (19).

### ZIKV_Natal_ infection of A. aegypti mosquitoes.

**FIG 4:**
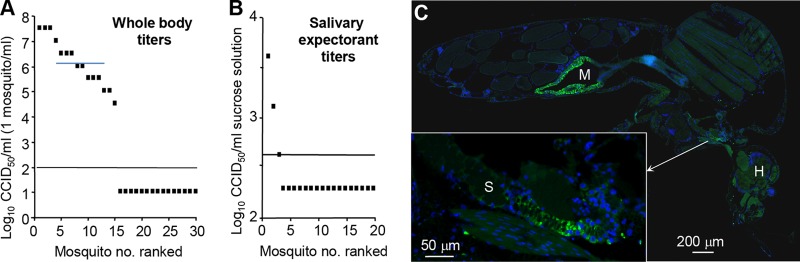
A. aegypti infection with ZIKV_Natal_. (A) A. aegypti mosquitoes were artificially fed blood meals containing ZIKV_Natal_, and after 14 days, the mosquitoes were homogenized in 1 ml of medium and viral titers were determined by CCID_50_ assay. The limit of detection was 2 log_10_ CCID_50_/ml of medium. (B) Same as for panel A, except that saliva was collected from a separate group of mosquitoes by allowing them to salivate into capillary tubes containing 10 μl of a sucrose-FBS solution. Titers were determined by CCID_50_ assay and represent the titers per milliliter of sucrose-FBS solution. The limit of detection was 2.6 log_10_ CCID_50_/ml. (C) Same as for panel A, except that mosquitoes were examined by fluorescence immunohistochemistry with 4G4, a flavivirus-specific antibody. M, midgut; H, head; S, salivary gland.

The main vector species in the Brazilian outbreak of ZIKV was A. aegypti (23–25). To determine whether ZIKV_Natal_ retains the ability to be transmitted by A. aegypti, standard artificial membrane feeding (with blood containing ZIKV_Natal_) was undertaken and mosquito infection was assessed by CCID_50_ assays after 14 days. Half of the successfully blood-fed mosquitoes contained detectable replicating virus, with whole-body titers ranging from 4.5 to 7.5 log_10_/CCID_50_/ml (one mosquito was macerated in 1 ml of medium) (Fig. 4A). Saliva was also collected from individual mosquitoes in a separate group of 20 (fed as described above and also tested 14 days postfeeding) by allowing the mosquitoes to salivate into capillary tubes containing a sucrose solution. Salivary expectorant from three mosquitoes (15%) contained detectable ZIKV, supporting the view that ZIKV_Natal_ retains the ability to be transmitted by A. aegypti. As mosquitoes expectorate an estimated 0.11 to 24 nl of saliva (26), the 2.6-log_10_ CCID_50_/ml titer (Fig. 3B) represents an estimated titer in saliva of 5.2 to 7.6 log_10_ CCID_50_/ml.

Immunofluorescent-antibody staining of whole mosquitoes (fed as described above, 14 days postfeeding) with 4G4 (pan-flavivirus anti-NS1 antibody) showed abundant staining of midgut cells, and in some mosquitoes, clear staining of the salivary glands was also evident (Fig. 3C). Taken together, these experiments argue that ZIKV_Natal_ remains mosquito transmission competent.

## DISCUSSION

Herein, we describe the *de novo* generation of ZIKV_Natal_ by a modified CPER protocol, with the resulting virus sequence identical (except for three synonymous nucleotide changes) to the published sequence obtained by next-generation sequencing of infected fetal brain tissue (7). ZIKV_Natal_ therefore represents the first ZIKV isolate without any passage history in cells and/or mice. In addition, as no virus isolate was obtained from the original brain tissues (7), this study also illustrates a novel pathway for obtaining a new virus isolate (next-generation sequencing of infected tissues, followed by *de novo* virus generation via CPER). Furthermore, ZIKV_Natal_ was able to infect fetal brains and cause IUGR in IFNAR^−/−^ fetuses and appears to retain the ability to be transmitted by A. aegypti mosquitoes. The CPER protocol thus provides a rapid method for obtaining an infectious flavivirus isolate from publically available sequence data without the need for international transport of infectious material.

A similar PCR-based protocol to generate African and Asian ZIKV isolates, as well as chimeric viruses, was recently reported that uses three DNA fragments covering the genome overlapping by ~70 to 80 nt (27). However, no joining of these fragments into circular DNA was performed. This may explain the relatively inefficient virus recovery observed in the study of Atieh et al., which required two passages to obtain viral titers of ~4 log_10_ CCID_50_/ml for the Asian isolate and ~5 log_10_ CCID_50_/ml for the African isolate. In contrast, the titers of passage 0 ZIKV_Natal_ generated in two different CPER and transfection experiments in our study were 5 × 10^5^ and 2.6 × 10^7^ PFU/ml, respectively, and after one passage of the former, the virus reached a titer of 1.25 × 10^8^ PFU/ml or 7.6 log_10_ CCID_50_/ml. The difference in the titers of viruses obtained in two different CPER assembly and transfection experiments is likely due to the differences in the processivity of the Q5 and PrimeSTAR GXL DNA polymerases, as well as the different cycling conditions used (more cycles and longer extension times for PrimeSTAR GXL DNA polymerase produced a higher virus titer), rather than the potential presence of mutations that could affect the infectivity of CPER-generated DNAs. Notably, CPER does not amplify DNA as conventional PCR would; it only doubles the amount of DNA by extending the gaps between annealed regions of fragments for each strand. Once polymerase reaches the next annealed region, it stops. Thus, unlike PCR, CPER is not an amplification process and therefore will not accumulate mutations as PCR could. In addition, both polymerases are high fidelity; hence, the introduction of different nucleotide changes in different independent experiments that could result in different infectivities of each CPER-generated DNA is unlikely.

A range of mouse models to study fetal infections by ZIKV have been reported (17). A range of strategies have been used to overcome the poor replication of ZIKV in WT mice, including (i) injection with anti-Ifnar1 monoclonal antibody (28, 29), (ii) infection via the intraperitoneal route later in pregnancy (E13.5) (30) or infection via the intravaginal route (18) (although fetal brain infection was only detectable by immunofluorescent-antibody staining), (iii) the use of SJL mice and very high doses of virus (22), (vi) direct intrauterine inoculation (31), and (v) infection of neonates (32). However, ZIKV does replicate in type I interferon response-deficient mice, with the use of IFNAR^−/−^ mice widely adopted (17). Infection of IFNAR^−/−^ mice with many ZIKV isolates is often highly symptomatic and lethal (17), although some recent isolates are showing reduced lethality in these mice (33, 34). A model using IFNAR^−/−^ females mated with WT males to produce IFNAR^+/−^ fetuses has been described, although maternal illness and demise and resorption of the majority of fetuses were noted (29). As ZIKV_Natal_ infection of IFNAR^−/−^ mice (>8 weeks old) via the s.c. route was asymptomatic and nonlethal, this system allowed infection of pregnant dams with minimal ethical concerns. Fetal head infections and the occurrence of IUGR in ZIKV_Natal_-infected IFNAR^−/−^ fetuses illustrate that this model recapitulates key elements of CZS.

In ZIKV_Natal_-infected IFNAR^−/−^ fetuses, when heads were infected, the corresponding placentas always had higher viral titers. In IFNAR^−/−^ mice, a range of placental cells appear to be infected (29). In humans, infection is largely restricted to Hofbauer cells, with placental trophoblasts thought to be protected from ZIKV infection by gamma interferon (35, 36), an activity that would clearly be absent in IFNAR^−/−^ mice. How, exactly, the virus traverses the placenta in humans remains unclear (3, 35–38). Although transplacental infection with other members of the flavivirus genus has only rarely been reported (3), perhaps of note, a virus in the pestivirus genus (family *Flaviviridae*), bovine viral diarrhea virus, can infect placental trophoblasts, leading to transplacental infection and congenital abnormalities (39, 40).

Our results support the view that ZIKV_Natal_ retains the ability to be transmitted by A. aegypti mosquitoes, with the difference in the percentage of mosquitoes infected and the percentage with infectious saliva broadly consistent with the reported rates for a variety of ZIKV isolates in these mosquitoes (23, 25). Previous studies have shown that Australian A. aegypti can transmit the ZIKA_MR766_ isolate (23), and our results now extend these findings to include a Brazilian ZIKV isolate associated with CZS. Australia has seen 128 imported ZIKV cases, 85 confirmed and the rest suspected (http://health.gov.au/internet/main/publishing.nsf/Content/ohp-vectorborne-overseas-acquired.htm; accessed 3 November 2017).

In summary, we have generated a new ZIKV isolate, ZIKV_Natal_, from sequence data and characterized the *in vitro* growth properties of the virus against those of the prototype, ZIKV_MR766_. Additionally, we have shown that it can recapitulate key aspects of CZS in a mouse model and can be transmitted by A. aegypti mosquitoes. ZIKV_Natal_ should thus find utility in future laboratory studies on ZIKV and provide a system for testing new interventions against ZIKV.

## MATERIALS AND METHODS

### Generation of ZIKV_Natal_ by CPER.

Seven dsDNA fragments covering the entire viral sequence (accession number KU527068) (Fig. 1A) were purchased from Integrated DNA Technologies, Inc. (Baulkham Hills, NSW, Australia), as Gblocks, and each was cloned into a separate pUC19 vector. For fragment 7, the last nucleotide (T) from the Gblocks gene fragment was omitted, as all flaviviruses end with TCT at the 3′ UTR, rather than the published TCTT sequence. These plasmids were grown in DH5α, the inserted sequences were verified, and the plasmids were then used to generate viral cDNA fragments by PCR (Fig. 1A). An additional PCR fragment (UTR linker) was generated from plasmid pUC19 containing the minimal CMV promoter, the first and the last 22 nt of the ZIKV_Natal_ sequence, the HDVr, and a pA signal (Fig. 1A). PCR fragments were generated with high-fidelity Q5 DNA polymerase and primer pairs that have complementary ends with a 24- to 30-nucleotide overlap (see Table S1). The resulting eight DNA fragments were then mixed in equimolar amounts (0.1 pmol each) and subjected to CPER with Q5 DNA polymerase (an initial 3 min of incubation at 98°C; 2 cycles of 30 s at 98°C, 30 s at 55°C, and 6 min at 72°C; and 10 cycles of 30 s at 98°C, 30 s at 55°C, and 72°C for 8 min) to generate circular DNA (Fig. 1A). In a separate CPER protocol, PrimeSTAR GXL DNA polymerase (TaKaRa) was used with the same eight PCR fragments (0.1 pmol each) but different cycling conditions (an initial 2 min of denaturation at 98°C; 20 cycles of 10 s at 98°C, 15 s at 55°C, and 12 min at 68°C; and a final extension for 12 min at 68°C.

10.1128/mSphereDirect.00190-17.2 TABLE S1 Primers used for PCR amplification of fragments used in CPER. Download TABLE S1, DOCX file, 0.01 MB. Copyright © 2017 Setoh et al. 2017 Setoh et al. This content is distributed under the terms of the Creative Commons Attribution 4.0 International license.

The CPER products were then transfected directly (without additional purification) into Vero cells with Lipofectamine LTX Plus transfection reagent (Life Technologies, Inc.), in accordance with the manufacturer’s instructions. At 6, 8, and 10 days after transfection, the culture supernatants were harvested. Day 10 supernatant (from the Q5 polymerase-generated CPER transfection) was expanded once in C6/36 cells (to produce passage 1 virus), aliquoted, and stored at −80°C prior to use in further experiments.

### RT-PCR for detection of viral RNA.

The cell culture supernatants collected at various time points posttransfection were subjected to RQ1 DNase (Promega) treatment to digest any CPER DNA left over from transfection; this was followed by viral RNA isolation with the NucleoSpin RNA Virus kit (Macherey-Nagel) and RT-PCR amplification with the SuperScript III One-Step RT-PCR System and platinum *Taq* DNA polymerase (Life Technologies, Inc.) with primers specific for the amplification of fragment 5 (30 min at 55°C; 2 min at 94°C; 35 cycles of 15 s at 94°C, 30 s at 55°C, and 2 min at 68°C; and a final extension for 5 min at 68°C). Total cell RNA was also isolated from transfected cells at day 10 after transfection and subjected to RT-PCR with the same primers and under the same cycling conditions. PCR amplification of viral RNA isolated from transfected cell supernatant and of total RNA from transfected cells was performed without RT with Q5 DNA polymerase (NEB) (3 min at 98°C; 35 cycles of 15 s at 98°C, 30 s at 55°C, and 2 min at 72°C; and a final extension for 5 min at 72°C) to confirm the lack of CPER DNA contamination.

### Immunofluorescent-antibody staining.

Vero cells were infected with the 10-day transfection supernatant, fixed with 100% acetone 3 days after infection, and stained with 4G4 (anti-NS1) (41) and 3G1 (anti-dsRNA) (42) antibodies and an Alexa Fluor 488-labeled goat anti-mouse IgG secondary antibody (Life Technologies, Inc.). Coverslips were mounted with ProLong Gold Antifade Mountant with 4',6-diamidino-2-phenylindole (DAPI; Life Technologies, Inc.), and immunofluorescence microscopy was performed with a Zeiss LSM710 confocal scanning microscope.

### Deep sequencing of viral RNA.

Viral RNA was isolated with the NucleoSpin RNA Virus kit (see above). RT-PCR amplicons (fragments 1, 2, 3, 4 plus 5, 6, and 7, Fig. 1A) for deep sequencing were generated from viral RNA isolated from 10-day culture fluid with the SuperScript III One-Step RT-PCR System and Platinum *Taq* DNA Polymerase (Life Technologies, Inc.). These represent the same pairs of primers that were used to generate PCR fragments for CPER, except that fragments 4 and 5 were combined into one amplicon. Libraries were prepared with the Nextera XT DNA Sample Preparation kit (Illumina Inc., San Diego, CA). The prepared library was sequenced on an Illumina NextSeq500 platform 2 × 150 bp PE run with V2 chemistry. Reads were mapped to the ZIKV_Natal_ genome (accession no. KU527068) with Bowtie 2 (1.1.2).

### Infection of cell lines for virus growth kinetics.

C6/36 cells (ATCC CRL-1660), Vero cells (CCL-81), A549 cells (ATCC CCL-185), WT MEFs, and IFNAR^−/−^ MEFs (43) were infected with passage 1 of a C6/36-derived stock of ZIKV_Natal_ or a C6/36-derived stock of ZIKV_MR766_ at the MOIs indicated, and 200 μl of culture supernatant was collected from each sample well at the postinfection times indicated. Six independent experiments were conducted with each cell line, with the exception of WT MEFs (MOI of 0.1), IFNAR^−/−^ MEFs, and C6/36 cells (*n* = 3). ZIKV_Natal_ titers were determined by standard plaque assay on Vero cells. Briefly, Vero cells were seeded into six-well plates and infected with 10-fold serial dilutions of virus samples for 1 h at 37°C, after which 2 ml of 0.75% low-melting-point agarose in Dulbecco’s modified Eagle’s medium supplemented with 5% fetal bovine serum was overlaid onto the cells and allowed to solidify before incubation at 37°C in 5% CO_2_. At 5 days postinfection, the cells were fixed with 4% formaldehyde for 30 min at room temperature. The agar overlay was then removed, and the fixed cells were stained with 0.2% crystal violet solution and the plaques were counted.

### Ethics statement.

All mouse work was conducted in accordance with the Australian Code for the Care and Use of Animals for Scientific Purposes, as defined by the National Health and Medical Research Council of Australia. Animal experiments were approved by the QIMR Berghofer Medical Research Institute animal ethics committee.

### Mice and ZIKV infection.

C57BL/6J mice were purchased from the Animal Resources Centre (Canning Vale, WA, Australia). IRF7^−/−^ mice were generated by T. Taniguchi (University of Tokyo) and provided by M. S. Diamond (Washington University School of Medicine, St. Louis, MO). IFNAR^−/−^ mice were provided by P. Hertzog (Monash University, Melbourne, VIC, Australia). The latter mice were on a C57BL/6J background (44, 45).

ZIKV_MR766_ (ATCC VR-84) stocks were prepared in low-passage-number C6/36 cells, aliquoted, and stored at −80°C (titer, 8 log_10_ CCID_50_/ml). Mice were infected s.c. at the base of the tail or i.p. in 50 μl of medium with the ZIKV doses indicated. A scorecard system was used to evaluate animal wellbeing and included scores for posture, mobility, swelling, fur ruffling, hind leg weakness, and injection site reactions. Mice were euthanized with CO_2_ when ethically defined endpoints were reached.

### ZIKV CCID_50_ assays.

ZIKV CCID_50_ assays for viremia and tissue titers were performed as previously described (46, 47), with minor modifications. Briefly, serum or supernatants from tissues or mosquitoes (bead macerated in medium) were collected and titrated in duplicate in 10-fold serial dilutions on low-passage-number C6/36 cells (ATCC CRL-1660). After 5 days, 50-μl volumes of supernatants were individually transferred onto parallel plates (i.e., A1 to A1, A2 to A2 … H12 to H12) containing low-passage-number Vero E6 cells (ATCC CRL-1586). After another 5 days, the plates were stained with crystal violet to visualize cytopathic effects. The titers were calculated by the method of Reed and Muench (50).

### A. aegypti infection.

An A. aegypti colony was established (at the insectory facilities of the QIMR Berghofer Medical Research Institute) from eggs of *Wolbachia*-free adult females collected at Innisfail, Australia, in April 2016. Mosquitoes were reared as previously described (48). Adult 3- to 4-day-old A. aegypti mosquitoes were fed blood meals containing a 1:1 mixture of ZIKV_Natal_ (7.6 log_10_ CCID_50_/ml) and defibrinated sheep blood by membrane feeder as previously described (47). Fed mosquitoes were collected and kept for 14 days at 28°C and 75% humidity as previously described (47). The mosquitoes were then anesthetized with CO_2_ and ice and homogenized (1 mosquito/ml of medium) as previously described (47), and after centrifugation, ZIKV titers in supernatants were determined by CCID_50_ assays as described above. Fluorescence immunohistochemistry of whole mosquitoes was also done as previously described (47), with pan-flavivirus, anti-NS1 protein monoclonal antibody 4G4 (41). Saliva was also collected from individual mosquitoes in a separate group of 20 at 14 days postfeeding by allowing the mosquitoes to salivate into capillary tubes containing 10 μl of a solution containing 10% sucrose and 10% fetal bovine serum (FBS) for 20 min as previously described (49), and titers were determined by CCID_50_ assays.
