# Supplementary figures and images for: De Novo Generation and Characterization of New Zika Virus Isolate Using Sequence Data from a Microcephaly Case
Source: mSphere. 2017 May 17;2(3):e00190-17. doi: 10.1128/mSphereDirect.00190-17 (PMC5437134; doi:10.1128/mSphereDirect.00190-17)

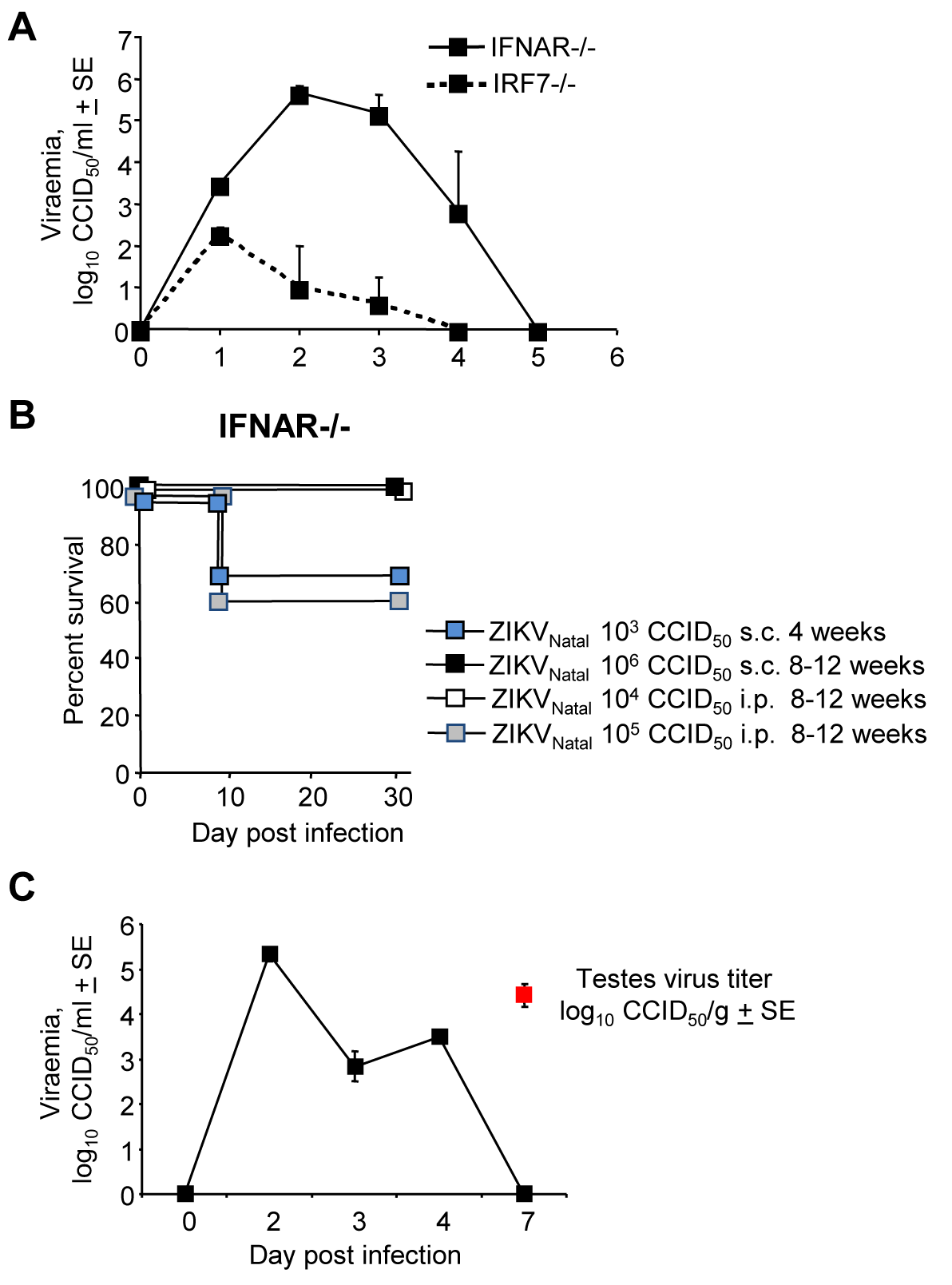

Supplement: FIG S1 [file sph003172289sf1.tif]
