# Supplementary material for: De Novo Generation and Characterization of New Zika Virus Isolate Using Sequence Data from a Microcephaly Case
Source: mSphere. 2017 May 17;2(3):e00190-17. doi: 10.1128/mSphereDirect.00190-17 (PMC5437134; doi:10.1128/mSphereDirect.00190-17)
Supplement: TABLE S1 [file sph003172289st2.docx]

| Fragment | Primer | 5’ – 3’ |
| --- | --- | --- |
| 1 | Forw | AGTTGTTGATCTGTGTGAATCAGAC |
|  | Rev | GTGAACGCTGCGGTACACAAGGAGTATG |
| 2 | Forw | CATACTCCTTGTGTACCGCAGCGTTCAC |
|  | Rev | CTTTCACGGGGTGTCCAATTAGCTCTGAAG |
| 3 | Forw | CTTCAGAGCTAATTGGACACCCCGTGAAAG |
|  | Rev | GACCCGTTTTCCAGCCTTTGTCAGAC |
| 4 | Forw | GTCTGACAAAGGCTGGAAAACGGGTC |
|  | Rev | GAACAAACTCTGGCGTCCATCCACCT |
| 5 | Forw | AGGTGGATGGACGCCAGAGTTTGTTC |
|  | Rev | TCCTGTTCCACCCCCACGTCTCTT |
| 6 | Forw | AAGAGACGTGGGGGTGGAACAGGA |
|  | Rev | ACTTGTCCGCTCCCCCTTTGGTCTT |
| 7 | Forw | AAGACCAAAGGGGGAGCGGACAAGT |
|  | Rev | AGACCCATGGATTTCCCCACACCG |
| UTRlinker | Forw | GGTGTGGGGAAATCCATGGGTCTGGGTCGGCATGGCATCTCCACC |
|  | Rev | GTCTGATTCACACAGATCAACAACTCGGTTCACTAAACGAGCTCTGCTTATATAG |

Supplementary Table 1. Primers for PCR amplification of fragments used in CPER
